# Snakebite Envenoming – A Combined Density Equalizing Mapping and Scientometric Analysis of the Publication History

**DOI:** 10.1371/journal.pntd.0005046

**Published:** 2016-11-07

**Authors:** David A. Groneberg, Victoria Geier, Doris Klingelhöfer, Alexander Gerber, Ulrich Kuch, Beatrix Kloft

**Affiliations:** 1 Institute of Occupational Medicine, Social Medicine and Environmental Medicine, Goethe University Frankfurt, Germany; 2 Institute of Occupational Medicine, Charité—School of Medicine, Germany; 3 Health Economics and Metrics, Department of Gynecology and Obstetrics, Goethe University Frankfurt, Germany; Faculty of Medicine, University of Kelaniya, SRI LANKA

## Abstract

Estimates suggest that more than 25,000 to 125,000 people die annually from snakebite envenomation worldwide. In contrast to this major disease burden, thorough bibliometric studies do not exist so far that illustrate the overall research activity over a long time span. Therefore, the NewQIS-platform conducted an analysis on snakebite envenoming using the Thomson Reuters database Web of Science. To determine and assess changes regarding the scientific activities and to specifically address the more recent situation we analyzed two time intervals (t). During the first time interval from 1900 to 2007 (t1) 13,015 publications (p) were identified. In the following period (2008–2016 = t2) 4,982 publications were identified by the same search strategy. They originate from 114 (t1) respectively 121 countries (t2), with the USA (p = 3518), Brazil (p = 1100) and Japan (p = 961) being most productive in the first period, and the USA (p = 1087), Brazil (p = 991) and China (p = 378) in the second period, respectively. Setting the publication numbers in relation to GDP/capita, Brazil leads with 92 publications per 10,000 Int$GDP/capita, followed by India with 79 publications per 10000 Int$GDP/capita (t1). Comparing the country’s publication activity with the Human Development Index level indicates that the majority of the publications is published by highly developed countries. When calculating the average citation rates (citations per published item = CR) mainly European countries show the highest ranks: From 1900–2007 Sweden ranks first with a CR = 27, followed by the Netherlands (CR = 24.8), Switzerland (CR = 23), Spain, Austria and the USA (CR = 22). From 2008 to 2016 the highest rate achieves Switzerland with a value of 24.6, followed by Belgium (CR = 18.1), Spain (CR = 16.7), Costa Rica (CR = 14.9) and Netherlands (CR = 14). Compared with this, the USA was placed at rank 13 (CR = 9,5).

In summary, the present study represents the first density-equalizing map projection and in-depth scientometric analysis of the global research output on snakebites and its venoms. So it draws a sketch of the worldwide publication architecture and indicates that countries with a high incidence of snakebites and a low economical level still need to be empowered in carrying out research in this area.

## Author Summary

Snakebite injury is a neglected tropical disease and lead to an enormous burden of disease in many parts of the world with about 25,000–125,000 estimated deaths. Therefore, research on this area of medicine is crucial since new diagnostic and therapeutic pathways may help to diminish disease burden. This study provides the first detailed landscape of the global snakebite research, which can be used by funding agencies and politicians to plan new programs. From 1900 to 2007 we found over 13,000 publications related to snakebites originating from over 110 countries, with the USA, Brazil and Japan being most active. The second period from 2008 until 2016 was characterized by 4,982 publications out of 121 countries. Here, the rank order was USA, Brazil and China. When efforts are analyzed from a socioeconomic perspective applying the GDP per Capita, Brazil and India take a lead position. The Human Development Index indicates that the vast majority of research is performed by highly developed countries. Closer investigations demonstrated that a large part of the research deals with scientific studies that used venom ingredients to unravel basic mechanisms of human physiology or to develop new pharmaceutical compounds. In summary, we here draw the first sketch of the overall global research architecture concerning snakebite envenoming. We found that countries with a high incidence of snakebites and a low economical level need to be empowered to carry out research in this area.

## Introduction

Snakebites envenomation is a neglected tropical disease and lead to an enormous burden of disease in many parts of the world [1–3]. Precise epidemiological data is missing but estimates suggest that 25,000–125,000 deaths and about 400,000 permanent disabilities are caused by snakebites annually [4–7]. The highest incidences were reported for (up to 500/100,000 inhabitants per year) Papua New Guinea, West Africa and Guinea [6,8,9]. Snakebites may also cause psychological morbidity [10]. However, these facts were previously largely neglected.

India plays a prominent role in snakebite epidemiology due to the vast size of the country and the number of inhabitants. However, potentially biased hospital-based statistics has led to widely ranging estimates of total annual snakebite mortality (1,300 to 50,000). Therefore, Mohapatra et al. calculated estimates of direct snakebite mortality from a national mortality survey using data from 123,000 deaths from 6,671 randomly selected areas between the years 2001 and 2003 [11]. They reported a number of 562 deaths (0.47% of total deaths), which was assigned to snakebites occurring mostly in rural areas (97%). This proportion was suggested to represent about 45,900 annual snakebite deaths nationally (99% CI 40,900 to 50,900) with higher rates in rural areas (5.4/100,000; 99% CI 4.8–6.0) [11]. The most reported annual snakebite deaths occur in the states of Uttar Pradesh (8,700 snakebite deaths), Andhra Pradesh (5,200 snakebite deaths), and Bihar (4,500 snakebite deaths) [11]. In view of this underestimation, snakebites need to be considered a neglected problem in twenty-first century India and South Asia in general [2,12].

Despite this substantial global burden, snakebite injuries have received only little attention from governmental and non-governmental institutions all over the world, the pharmaceutical industry, and public health authorities and advocacy groups. Also, research funding and resources for health programs are on a low level in comparison to other diseases. In this respect, stakeholders and decision-makers have a huge requirement for scientifically validated recommendations that are still not sufficiently available. The inclusion of snakebites in the WHO list of Neglected Tropical Diseases (NTDs) as well as the development of project and initiatives by the WHO may help to improve global awareness.

The fight against snakebite-related diseases is also crucially dependent on research funding. Usually, the funding allocation processes get improved by adding scientometric features to the election processes. Unfortunately, there is a lack of basic information about snakebite research. Therefore, we carried out a combined density equalizing mapping and scientometric analysis. The here presented patterns of the research efforts and the publication output empower scientific institutions, planners, and stakeholders to allocate research funding better, and support the most affected regions.

## Methods

### NewQiS-platform

The present study is part of the NewQiS-platform, which uses novel visualizing techniques in combination with new and established scientometric analysis tools [13,14].

### Density-Equalizing Mapping

The principle algorithm of density-equalizing map projections (DEMP) was reported by Gastner and Newman [15] and incorporated in NewQIS-studies [16]. In brief, software applying these algorithms is used to determine correlations and differences between countries that are publishing on snakebite-research. The method resizes countries proportionally according to a predefined variable. In this process, the nation with the highest value of the analyzed parameter is depicted largest on the associated map, whereas regions without or with a very low value are proportionally scaled down.

### Data source

All analyzed data was retrieved from the Thomson Reuters online-database ‘Web of Science’ (WoS). The evaluation period was divided into two intervals. The first time frame was limited to the period between 1900 and 2007 (t1) in order to assess a closed and defined interval to reach a historical bibliometric evaluation. This time period starts after the epochal invention, and introduction into clinical medicine, of antivenom treatment for snakebite envenoming by Albert Calmette [17] and Vital Brazil at the end of the 19^th^ century [18], and ends with the publication of the key conceptual snakebite advocacy paper by Gutiérrez et al. in 2006 [19]. Publications from the subsequent years up to the time of analysis (2008–2016) were addressed separately (t2) because this timespan has been marked by a fresh interest in snakebite injuries and related advocacy initiatives which have led to substantial changes and new hope for this neglected field of research. These included, for example, events like WHO regional and bi-regional workshops leading to the publication of ‘WHO Guidelines on the production and use of snake antivenom immunoglobulins’ [20] with an extensive associated internet database of venomous snakes and antivenoms [21], a revised edition of ‘WHO-SEARO Guidelines for the management of snakebite in South and Southeast Asia’ [22], ‘WHO Guidelines for the management of snakebite in Africa’ [23], the ‘Global Issues in Clinical Toxicology’ conference in 2008 with the formation of the ‘Global Snakebite Initiative (GSI)’ [24], several regional conferences in Africa with the foundation of the ‘Société Africaine de Venimologie / African Society of Toxicology’ [25], and a series of influential high-profile articles [26–29] that have helped generate awareness and a re-emerging interest for snakebite envenoming in professional societies as well as funding agencies.

### Data categorization

The methodology of data categorization was performed as reported also in previous NewQIS-publications [30–32]. Using this method, the retrieved bibliometric data has been categorized according to various parameters (e.g. publication countries, publication year, publishing authors, published document type, and assigned subject categories). Afterwards it has been transformed into a database format. Data adjustments led to more meaningful findings by the correction of author’s data. This has proved necessary because the software-supported assignment of bibliometric data is faulty and cannot be evaluated accurately when e.g. the country or origin or the author’s specifications are erroneous or outdated.

### Search strategies

The following composed query term has been applied via the WoS Topic-Search: ((snake*) AND (venom* OR envenomation* OR poison* OR toxin* OR antidote* OR antiserum* OR antivenom*)) OR ((bit* OR venom* OR envenomation* OR poison* OR toxin* OR antidote* OR antiserum* OR antivenom*) AND (viper* OR elapid* OR colubrid* OR atractaspid* OR naja OR cobra* OR crotalus OR rattlesnake* OR bothrops OR lancehead* OR agkistrodon OR moccasin* OR bungarus OR krait* OR echis OR saw-scaled viper* OR dendroaspis OR mamba* OR trimeresurus OR asian pit viper* OR bitis OR puff adder* OR notechis OR tiger snake* OR oxyuranus OR taipan* OR lachesis OR bushmaster* OR cerastes OR horned viper* OR dispholidus OR boomslang* OR hydrophii?ae OR sea snake*)). The Boolean operator connects the individual terms as disjunction. The asterisk is used as a placeholder for several characters, while the question mark stands for one letter only.

### Analysis of origin / language

The information about the address of the author’s institutions was analyzed in order to determine the country of origin.

The interpretation of international publications is based on added up values. Herewith every national contribution has been counted separately, so that the overall publication sum of the country-specific analysis is much above the actual amount of the retrieved articles.

Countries that are no longer existing (e.g. USSR, Yugoslavia), or that have been formed later (e.g. Germany from the former FRG and GDR) have been compared with an updated list of countries and self-governed regions, and corrected respectively.

### Citations rate

Additionally, chronological analyses have been performed. By this procedure, it was possible to assess the total number of publications and citations per publication year until the date of analysis. Also, the average citation per year was computed for years with at least 30 published items. This threshold was implemented for all citation rate analyses to reduce the impact and the consequent bias of both years in which very few articles have been published or of countries with a very number of publications.

### Social, economic and epidemiologic indices

The Human Development Index (HDI) of the Human Development Report (HDR) of the UN Development Project (UNDP) was used to relate the research activity to social and economic data [33].

Also, the GDP/capita in 10 thousand Int$ was used. Due to the exclusion of Taiwan from the World Bank, the data was retrieved from the World Factbook that includes Taiwan [34,35]. Epidemiologic indices were used from the estimates of Kasturiratne et al. [4].

### Analysis of country cooperations

An analysis of international cooperations was carried out to assess research networks [36][35]. In brief, a bilateral cooperation between two countries was defined when at least one author originates from one country and at least one other author from a second country. A matrix with all identified countries was set up and filled with the corresponding values for the cooperation for each pair of countries. A second software module was used to translate the matrix and to transform the figures into vectors.

## Results

### General parameters

In t1 a total number of 13,015 publications were identified, and the second evaluation period (t2) delivered 4,982 publications. A strong increase between the years 1990 and 1991 is due to the inclusion of abstracts and keywords in the search mode of WoS. In the first evaluation period (t1) 87% of the publications were attributable to one or more countries, viz. 11,323 publications, whereas in t2 only 1,4% was not attributable, so that 4,913 publications were assigned to one or more countries. In total, 114 (t1), respectively 106 countries (t2) contributed to the overall publication output. Ninety-four percent (t1) and alternatively 97% in t2 of all publications were written in English. The most common non-English articles were published in French (2%), Russian (0.97%), and German (0.96%) in timespan t1, while in t2 French (0,64%), Spanish (0,62%) and Portuguese (0,54%) were the most frequent non-English languages. Despite the already high level of English-written publications in t1, this proportion has even more increased in t2.

### Density equalizing mapping of research activity

The USA was the leading country concerning research output with a total of 3,518 published items representing 31% of the attributable publications (t1), respectively 1,087 publications (22%) in t2. On second position in both periods, Brazil was listed with 1,100 publications (10%) in t1 and 991 publications (20%) in t2, respectively.

**Fig 1:**
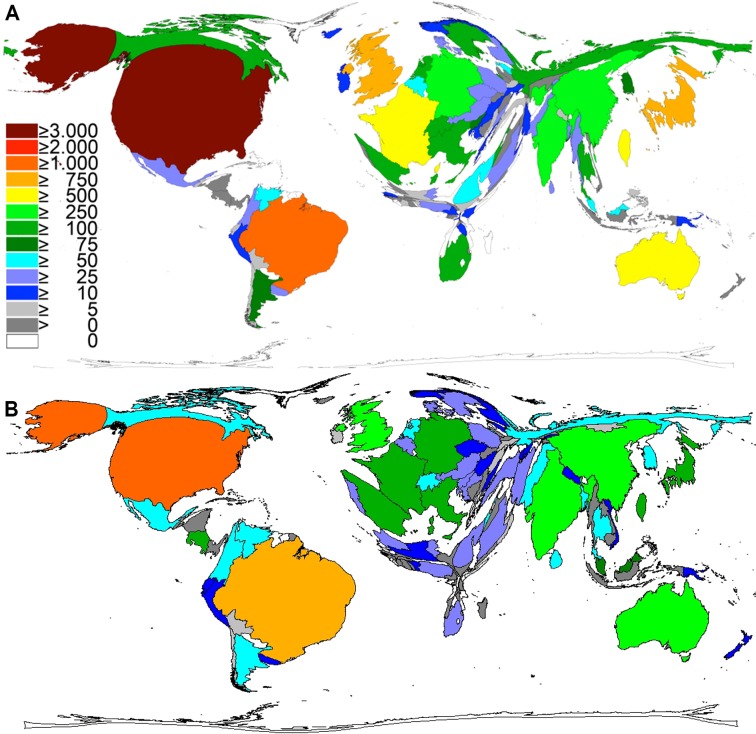
Global publication output of snakebite-related research. Density-equalizing map projection. A) 1900–2007. B) 2008–2016.

**Fig 2:**
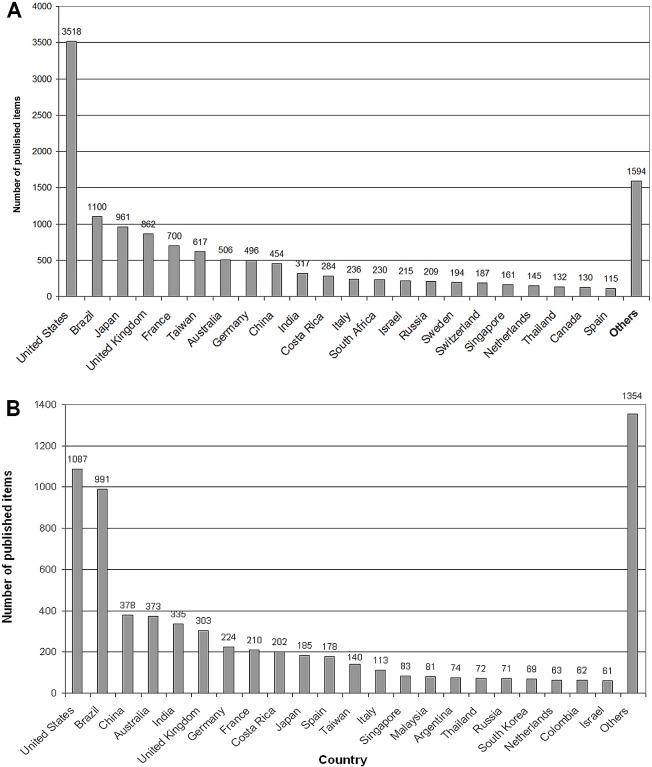
Global number of snakebite-related publications of the most publishing countries. A) 1900–2007. B) 2008–2016.

In t1, these two countries were followed by Japan with p = 961 (9%), the UK with p = 862 (8%), France with p = 700 (6%), Taiwan with p = 617 (5%), Australia with p = 506 (4%), Germany with p = 496 (4%), China with p = 454 (4%), and India with 317 publications (3%). Costa Rica (p = 284; 3%), Italy (p = 236; 2%), South Africa (p = 230; 2%), Israel (p = 215; 2%), and Russia (p = 209; 2%). Sweden (p = 194; 2%), Switzerland (p = 187; 2%), Singapore (p = 161; 1%), the Netherlands (p = 145; 1%), Thailand (p = 132; 1%), Canada (p = 130; 1%) and Spain (p = 115; 1%) have more than 100 published items each. The other 92 countries, with less than 100 publications each, accounted for 1594 publications in total (Fig 1A and Fig 2A).

In the second evaluation period (t2) the third most active country was China with 378 items (7.7%). The next countries were Australia with p = 373 (7.6%), India with p = 335 (6.8%), UK with p = 303 (6.2%), Germany with p = 224 (4.5%), France with p = 210 (4.3%), Costa Rica with p = 202 (4.1%), Japan with p = 185 (3.8%), Spain with p = 178 (3.6%), Taiwan with p = 140 (2.8%), and Italy with p = 113 (2.3%). Less than 100 publications were published by another 108 countries (Fig 1B and Fig 2B).

### Research output in relation to incidence and economic characteristics

When comparing the research activity to incidence of snakebites and economic data of the publishing countries, a two-fold result was obtained (Tab. 1): Due to the low incidence, the country with the highest research output (USA) was increasing its distance to the second ranked country, publications per snakebites per 100,000 inhabitants in the US versus 356 for Japan. Other countries with low incidences such as the UK, or France and Germany also raised in this ranking. By contrast, when relating the output data to GDP/capita, Brazil with 92 publications per 10,000 Int$ GDP/capita was ranked number one. Second was India with 79 publications per 10,000 Int$ GDP/capita.

### Relation of publications to WHO world regions and their incidences and mortality rates and HDI levels

**Fig 3:**
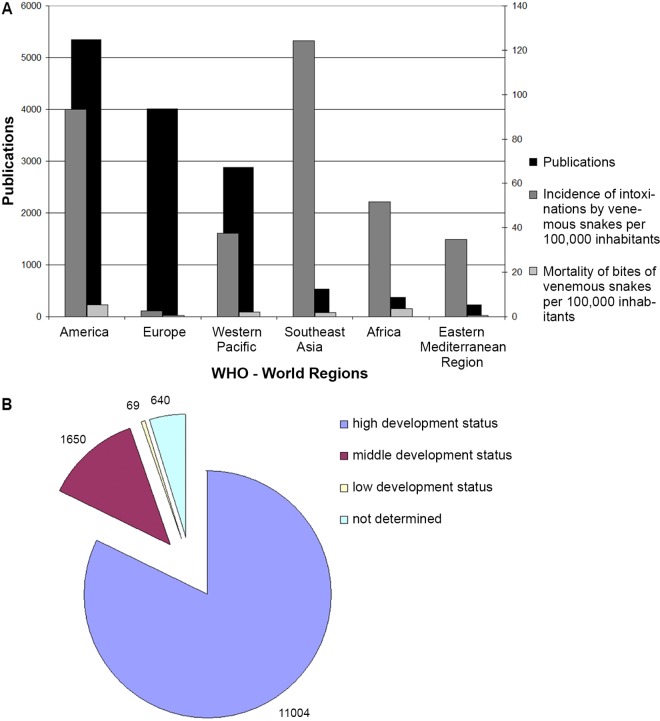
Comparison of publication activity on snakebite envenoming and snake venoms. A) Comparison with the incidence and mortality estimates of the six WHO regions. B) Comparison with the level of development (Human Development Index).

The adjustment of the research activity data to the six WHO regions (WHO African Region, WHO Region of the Americas, WHO South-East Asia Region, WHO European Region, WHO Eastern Mediterranean Region and WHO Western Pacific Region) led to the following ranking: The WHO Region of the Americas was leading with p = 5,343 (47%) attributable publications, followed by the WHO European Region with p = 4,015 (36%) and the WHO Western Pacific Region (Fig 3A). By contrast, the snakebite incidence was higher in the WHO South-East Asia Region.

When adjusting the output data to the HDI level according to the Human Development Report, in the majority of the publications (p = 11,004, 97%) a highly developed country was at least collaborating (Fig 3B). By contrast, only in 1,650 (14.6%) of the publications, a country with a medium HDI level is participating and only in 69 publications, a low level country is participating.

### International collaborations

**Fig 4:**
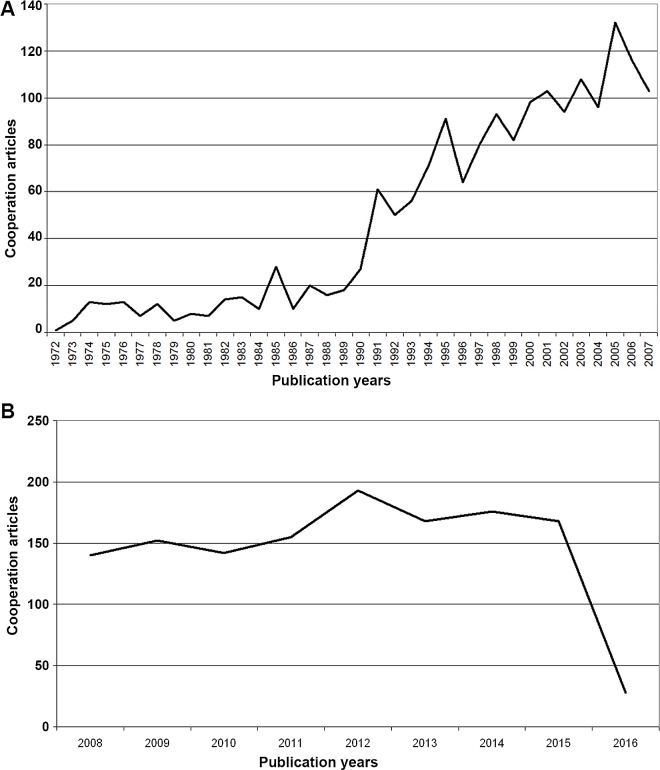
Number of collaboration articles on snakebite envenoming and snake venoms. A) 1900–2007. B) 2008–2016.

**Fig 5:**
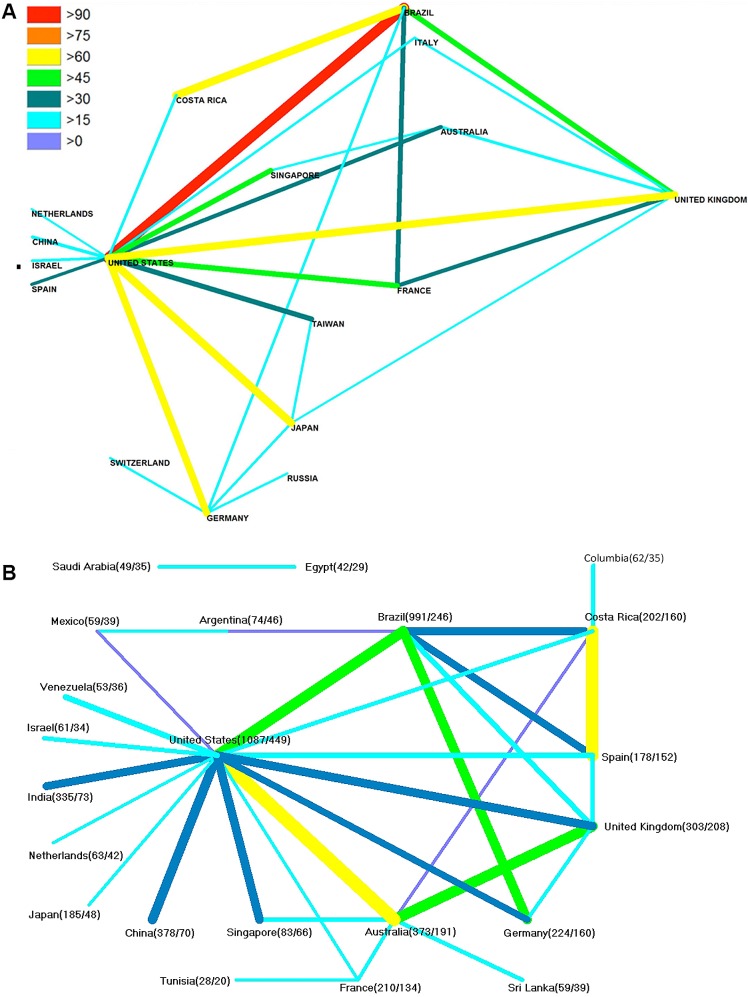
International collaboration network of countries working on snakebites and snake venoms. Threshold = 15 collaboration articles. A) 1900–2007. B) 2008–2016.

The number of international collaborations between two or more countries increased steadily since 1972 (Fig 4A) to the end of period t1 in 2007. Also, between 2008 and 2016 (t2) the trend towards an increasing number of collaborations has been remaining–with exception of 2016, due to the missing values of this incomplete evaluation year (Fig 4B). The maximal levels were reached with 132 (t1 = 2005) and 193 collaborations (t2 = 2012). In total, 1,739 cooperation articles (CA) were identified from 1972 to 2007 (t1) and 1,322 until 2016 (t2). Out of them, CA = 1,480 in t1 (t2: CA = 997) are a result of a bilateral cooperation; t1: CA = 211, t2: CA = 238 publications are trilateral. Four countries collaborated in CA = 38 (t1) and CA = 57 (t2). The USA participated in CA = 843 until 2007 (t1) and in CA = 633 in t2, the UK in CA = 456 (t1) and CA = 358 (t2), Brazil in CA = 365 (t1) and CA = 342 (t2), France in CA = 352 (t1) and CA = 226 (t2), and Germany in CA = 303 (t1) and CA = 278 (t2). Forty cooperation country pairs were found with USA and Brazil cooperations being highest in number (CA = 92) until 2007 (Fig 5A). In t2 (2008–2016) the bilateral works of the USA and Brazil ranked only third with an amount of CA = 52. Here, the highest number has been reached by the collaboration of Australia and the USA with CA = 63 common publications (Fig 5B).

### Citation analysis

**Fig 6:**
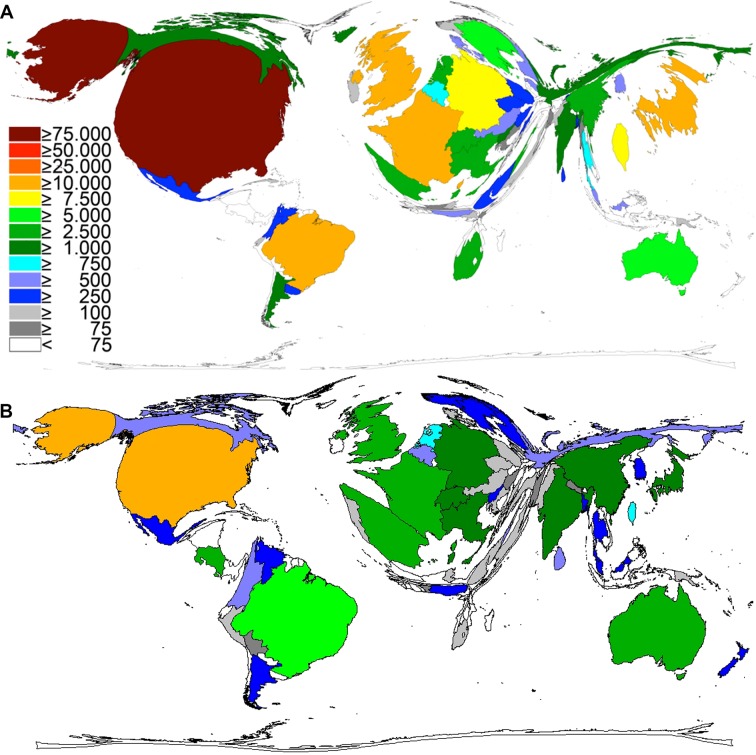
Global number of citations of snakebite and snake venom related publications. Density-equalizing map projection. A) 1900–2007. B) 2008–2016.

In respect to the number of citations (c), the USA led in both time intervals with t1: c = 75,614 and t2: c = 10,420. Until 2007 (t1) the UK is ranked 2^nd^ with c = 17,908, followed by Japan (c = 17,606), France (c = 14,612) and Brazil (c = 12,028) (Fig 6A). In contrast, the second evaluation period (2008–2016) showed another continuing order. The second most cited country in t2 was Brazil with c = 6519, followed by Australia (c = 4,209), the UK (c = 3,868) and Costa Rica with c = 2,998 (Fig 6B).

**Fig 7:**
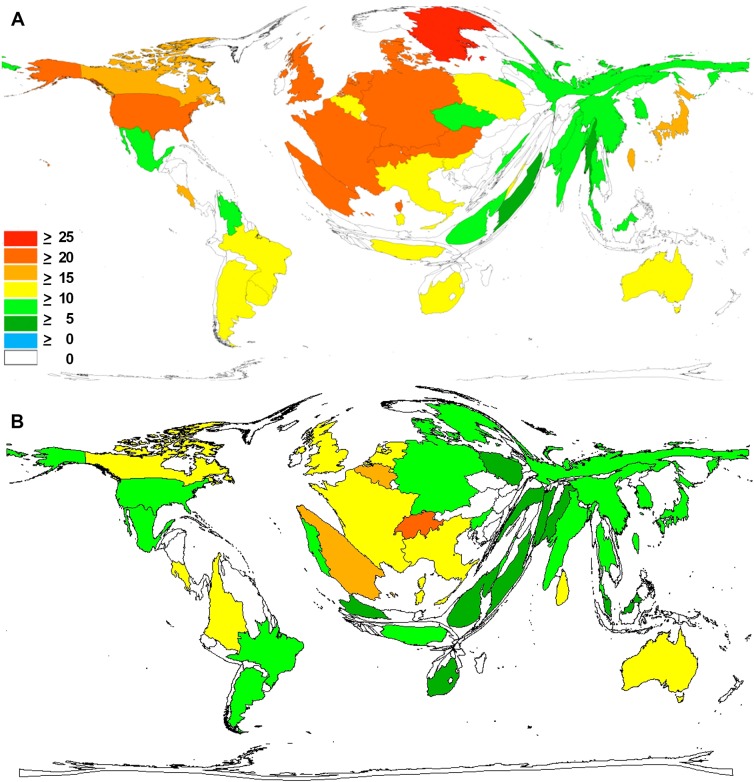
Average citation rate of snakebite and snake venom related publications. Density-equalizing map projection. A) 1900–2007. B) 2008–2016.

When calculating the citation rate per published item of each country in a DEMP with a threshold of at least 30 publications per country, a different global research landscape has been obtained with European countries taking the lead position. In the first time span (t1), Sweden ranked number 1 with a citation rate (CR) of 27, followed by the Netherlands (CR = 24.8), Switzerland (CR = 23), Spain and Austria (CR = 22). At position six, the USA came up with a citation rate of 22, followed by other European countries (France CR = 21, the UK CR = 21, Denmark CR = 21 and Germany CR = 20). At position 11, non-European countries followed with Costa Rica (CR = 19), Japan (CR = 18), Canada (CR = 17) and Taiwan (CR = 16) leading the field. Brazil has a citation rate of 11 citations per publication (Fig 7A).

The analysis of the citation rate of the second time period (t2) revealed a different picture. Here, Switzerland had the leading position with CR = 24.6, followed by Belgium (CR = 18). Placed next were Spain (CR = 16.7), Costa Rica (14.8), the Netherlands (CR = 14), UK (CR = 12.8), France (CR = 12.2), Australia (CR = 11.3), Italy (CR = 11.2) and Colombia (CR = 10.9). The USA was ranked only 13^th^ with a rate even under 10 (CR = 9.6) (Fig 7B).

### Institutional output

**Fig 8:**
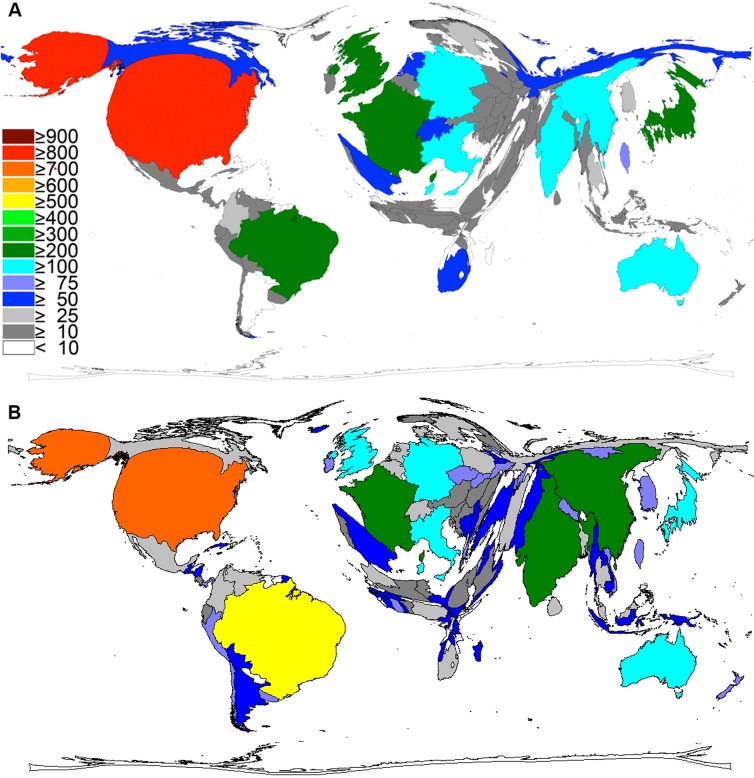
Number of institutions publishing on snakebite and snake venom research. Density-equalizing map projection. A) 1900–2007. B) 2008–2016.

The 13,014 identified publications of t1 were published by a total of 3,922 institutions (i). When analyzing the number of institutions per country, the USA were leading with i = 896 institutions, followed by Japan with i = 284, Brazil with i = 283, France with i = 259 and the UK with i = 217 (Fig 8A).

**Fig 9:**
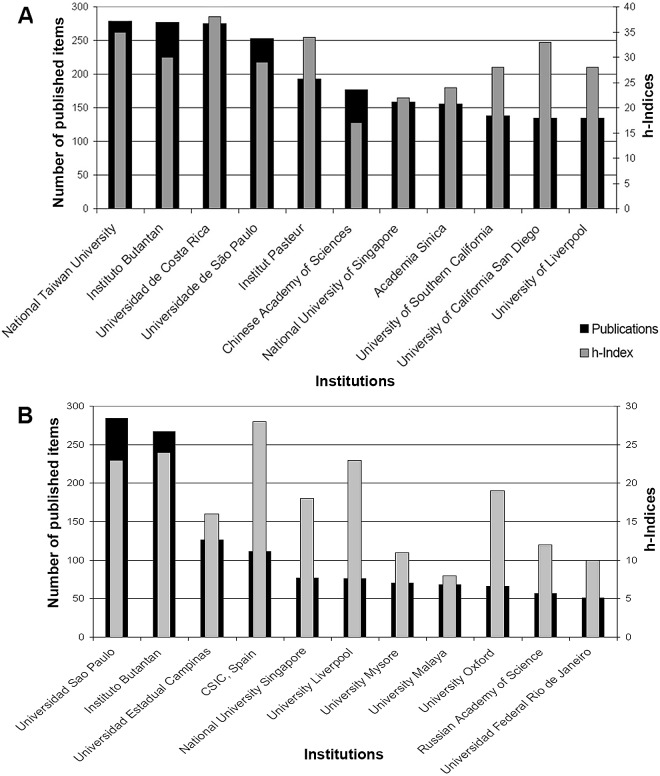
Leading institutions concerning output and modified h-index of snakebite related research. A) 1900–2007. B) 2008–2016.

The analysis of the institutions participating in t1 showed a range of 1 to 279 publications per single institution. The National Taiwan University was ranked number one with 279 publications, followed by the Brazilian Instituto Butantan with p = 277 and the Universidad de Costa Rica (Instituto Clodomiro Picado) with p = 275. The highest modified country-specific h-index within the analyzed set of p = 11,302 was found for the Universidad de Costa Rica with a value of 38 (i.e. 38 publications that were at least cited 38 times) (Fig 9A); all of these from the university’s Instituto Clodomiro Picado.

In t2 there were 705 institutions in the USA publishing on snakebites, followed by Brazil with i = 540, China (i = 291), India (i = 284), and France (i = 158) (Fig 8B). The range of publications per institution varied from 1 to 284 snakebite related publications (University of Sao Paolo, Fig 9). As in t1, the second most active institution was the Brazilian Instituto Butantan with p = 267 from 2008 to 2016 (t2). Another Brazilian institution followed with p = 126, the Universidad Estadual Campinas. The highest snakebite-specific h-index in t2 was achieved by the Spanish National Research Council CSIC (‘Consejo Superior de Investigaciones Científicas’ Fig 9B).

## Discussion

*PLOS Neglected Tropical Diseases* has published in the past years numerous studies related to the field of snakebites [37,38]. They range, for example, from neurotoxicological aspects [39], immune responses [37], phylogeny, venom composition of diverse species [38], the analysis of geographical information [40] to basic science issues [41–43] and public health [44]. Despite the burden of disease caused by snakebite envenoming and the need for increased transnational and national funding, there has been no in-depth scientometric analysis of this topic so far. Therefore, we used the NewQIS-platform methodology to conduct a combined DEMP and scientometric study on publications related to snakebites until 2016.

Bibliometric tools are commonly used to dissect research profiles also for neglected tropical diseases [45–47] and using such approaches, a number of methodological issues need to be discussed: First, it has to be considered that the analysis of snakebite related articles in the present study cannot be regarded as completely representative of global snakebite research activity, since the data was retrieved from only one database (Web of Science), denoting a potential bias. However, we used the WoS since this database enabled us to assess also qualitative aspects (citations). Whereas the WoS is among the largest global biomedical databases, there are of course still publications, which cannot be traced by the use of this system. Nonetheless, it can be hypothesized that the present findings represent common trends in the research on snakebite and snake venoms and toxins. Second, the employed indicators that refer to the significance of the publications in the scientific community (number of citations, citation rate) need to be regarded critically and therefore, the data should not be over interpreted concerning research quality, as indicated by numerous previous articles [26–28]. In fact, assessing the quality of research is only possible by advanced meta-analysis using, for example, Cochrane approaches [48]. Insofar, the findings of the citation-related analyses refer to the resonance and the attention that the publications gained in the scientific community. This is certainly not always connected with the publications’ quality, but it indicates the interest with which scientific peers take them into account. Third, the research output data was related to epidemiological estimates [4] which do not necessarily display the actual case numbers in different countries exactly [4,11,49]. Fourth, there is a language bias present within the biomedical databases. Publications written in English have a higher chance of being included [50]. Also, established journals are listed more frequently than novel journals although the latter may have the same quality standards [51]. Therefore, the Matthew effect needs to be considered [52]: The communication systems in science are directed towards a reward for highly productive and renowned journals, scientists and institutions which leads, for example, to a pyramidal citation scheme.

Over two hundred years ago, the Scottish surgeon Patrick Russell published a work entitled "An Account of Indian Serpents Collected on the Coast of Coromandel" (1796) which can be regarded as one of the first scientific publications that point to the need of differentiating between venomous and non-venomous snakes and the necessity to develop therapeutic options for snakebite envenoming [53]. About one hundred years later, Léon Charles Albert Calmette developed the first snake antivenom at the Institute Pasteur de Saigon [54,55].

We therefore decided to start the analysis at the beginning of WoS entries in 1900. Thenceforward, we analyzed two different time intervals. The first time frame was closed in 2007 due to the time that is needed for each publication to show their highest impact measured as Cited Half-Life. This value has been introduced by the originator of the JCR (Journal Citation Report) Eugene Garfield, meaning the time span that is necessary to get at least 50% of the citations [56]. Regarding the biomedical literature the mean Cited Half-Life can be considered up to eight years with a growing annual trend [57]. Some journals publishing on snakebites reaches even a Cited Half-Life of more than ten years (e.g. Archives of Ophthalmology). The second evaluation time frame was set from 2008 to 2016 to include also the more current research outputs up to now.

The analysis shows that from 1900 to 2016, the yearly published amount of snakebite related publications increases from 5 (1900) to 677 (2012). This exemplifies the increase in scientific activity in this scientific area but is also related to the general bibliometric principle that research articles usually double within a time frame of 10 to 20 years [58]. The years after the maximum in 2012 show a slight decrease to 659 publications in 2014. The even lower publication numbers in 2015 (p = 586) and 2016 (p = 82) are due to the fact that on the one hand not all accepted publications are already listed (2015) and on the other hand the year 2016 is not terminated at the time of analysis, so that only a part of the real publication output of these years is taken into account.

Maxima of publications numbers in single years are often related to important scientific findings and usually occur 2–5 years after the publication of the important finding. In this respect, the observed maximum in 1978 (198 publications) is most likely related to the development of Captopril from the venom of the South American Jararaca (*Bothrops jararaca*). Also, the maximum in 1985 (281 publications) is most likely related to the finding of dendrotoxin in 1980, which was isolated from Mamba venom.

The strong increase in 1991 is not due to a scientific but to a methodological reason caused by the implementation of the Topic-search tool of WoS.

When analyzing the contribution of single countries, neither the estimated incidence (1.7 snakebite injuries per 100,000 inhabitants) nor the mortality (0.01 per 100,000 inhabitants) but the general standing of the USA as the world leading country concerning research activity points to the large gap in overall snakebite-research activity. This indicates that countries with a high incidence of snakebites and a low economical level need to be empowered to carry out research.

In striking contrast to other scientometric studies that addresses diseases such as gout [59], silicosis [31], or infectious diseases including influenza [35], or hepatitis B [60], the global ranking of snakebite research activity is different to the usual picture with the USA being followed by the UK, Germany or Japan: This usual pattern has also been found in a study analyzing over 5.5 million publications on the global publication activity within the following 21 organ systems: Brain, heart, artery, vein, lung, muscle, eye, nose, ear, throat, neck, skin, breast, stomach, intestine, pancreas, kidney, genital, hormone, arm, feet. Here, an almost uniform pattern was present for every single organ. The USA was ranked first in every of the 21 organ systems. Number two and three concerning research output were either the UK, Japan, Germany or France. Interestingly, a dichotomy was present between Western countries such as the USA, UK or Germany and Asian countries such as Japan, China or South Korea concerning research focuses. Western countries each had the following ranking concerning research activity: the most frequently focused organ was the heart. By contrast, the Asian countries had the liver as number one organ of interest.

In contrast, the current analysis shows that Brazil, a country with a relatively high incidence and mortality and with a rather low GDP contributed with the second highest research activity. The Brazilian publication performance corresponds with the findings of another neglected disease study on yellow fever [61]. Here, a recent NewQIS-study identified a total of 5,053 yellow fever-associated publications, which were published by 79 countries.

The difference of the overall numbers of publications between yellow fever and snakebite related publications is most probably caused by the inclusion (search term) of studies that use venom ingredients to address basic mechanisms of human physiology and to develop novel pharmaceutics.

Until now basic research on snakebites and venoms is more or less privileged to high-income countries, firstly, because they have the means for high tech research and secondly, there are no or only very little problems with snake envenomation. This is obviously the opposite in middle-income and low-income countries. The lack of resources limits the research on snakebite envenoming in these countries. Nevertheless, the emerging nation Brazil established–even enhanced–an important role in snakebite research. Also, as shown here, other countries from South and Middle America, like Costa Rica and Colombia, have appeared in the recent decade on the global landscape of snakebite research. Hence, it should be requested that other directly affected low and middle nations are also integrated to the worldwide research networks.

## Conclusions

The present study represents the first density-equalizing mapping and scientometric analysis of the worldwide research activities on the subject of snakebites and draws a sketch of its overall global research architecture. The ten and more years old calls for global snake-bite control and procurement funding needs to be re-emphasized [62].
